# Supplementary material for: Identification of Predictive Factors for Overall Survival and Response during Hypomethylating Treatment in Very Elderly (≥75 Years) Acute Myeloid Leukemia Patients: A Multicenter Real-Life Experience
Source: Cancers (Basel). 2022 Oct 6;14(19):4897. doi: 10.3390/cancers14194897 (PMC9564161; doi:10.3390/cancers14194897)
Supplement: Supplementary file 1 [file cancers-14-04897-s001.zip › cancers-1925132-supplementary.pdf]

Supplemental Table S1. Univariate Logistic Regression Models for Response (CR)

**Variable**	**Predictive Factor**	
	**p value**	**OR (95% CI)**
**Age at diagnosis**		
(75-80 years vs ≥80)	*0.006*	2.99 (1.37-6.53)
**Sex**		
(female vs male)	0.408	1.30 (0.70-2.44)
**ECOG PS**		
(<2 vs ≥2)	*0.034*	2.15 (1.06-4.27)
**Type of AML**		
(s-AML vs de novo AML)	*0.005*	0.35 (0.17-0.74)
**eGFR**		
(<60 vs ≥60 ml/min/1.73m^2)	*0.013*	0.16 (0.04-0.67)
**CCI**		
(<3 vs ≥3)	*<0.001*	6.62 (3.22-13.62)
**BMI at diagnosis**		
(<25 vs ≥25)	*0.018*	2.26 (1.15-4.44)
**BM blast percentage**		
(≥50% vs <50%)	*<0.001*	2.18 (1.59-2.98)
**Infectious at AML diagnosis**		
(no vs yes)	*0.045*	2.56 (1.02-6.42)
**Time from diagnosis to the start of therapy**		
(<15 days vs 15—30 days)	0.639	0.84 (0.40-1.75)
(<15 days vs >30 days)	0.237	0.79 (0.53-1.12)
(15—30 days vs >30 days)	0.120	0.52 (0.23-1.19)
**Transfusion dependency at diagnosis**		
(yes vs no)	*0.050*	0.53 (0.28-0.99)
**Complex Karyotype**		
(yes vs no)	*0.014*	0.26 (0.09-0.75)
**ELN risk category**		
Adverse risk vs low-intermediate risk	*0.007*	0.78 (0.18-0.77)
**Type of HMA**		
(AZA vs DEC)	0.143	0.60 (0.30-1.19)

**Starting HMA dose**		
(reduced vs standard)	0.476	0.46 (0.06-3.85)
**Treatment-related complication**		
(yes vs no)	0.721	0.68 (0.08-5.74)

Abbreviation: AML, acute myeloid leukemia; AZA, azacitidine; BM, bone marrow; BMI, body mass index; CCI, Charlson comorbidity index; CI, confidence interval; DEC, decitabine; ECOG PS, Eastern Cooperative Oncology Group Performance Status; eGFR, estimated glomerular filtration rate; ELN, European Leukemia Net; HMA, hypomethylating agents**;** OR, odds ratio; s-AML, secondary AML

Supplemental Table S2. Univariate Cox Proportional Hazards Models for Overall Survival

**Variable**	**Predictive Factor**	
	**p value**	**HR (95% CI)**
**Age at diagnosis**		
(≥80 vs 75-80 years)	*0.002*	1.66 (1.21-2.30)
**Sex**		
(female vs male)	0.953	0.95 (0.71-1.28)
**ECOG PS**		
(≥2 vs <2)	*0.009*	1.64 (1.13-2.38)
**Type of AML**		
(s-AML vs de novo AML)	0.207	1.21 (0.89-1.64)
**eGFR**		
(<30 vs ≥60 ml/min/1.73m^2)	0.126	1.38 (0.92-2.01)
**CCI**		
(≥3 vs <3)	*0.030*	1.52 (1.04-2.33)
**BMI at diagnosis**		
(<25 vs ≥25)	0.121	0.79 (0.59-1.06)
**BM blast percentage**		
(≥50% vs <50%)	*<0.001*	1.97 (1.44-2.72)

**Infectious at AML diagnosis**		
(no vs yes)	0.204	0.79 (0.55-1.14)
**Time from diagnosis to the start of therapy**		
**(**<15 days vs 15—30 days)	0.167	1.29 (0.89-1.85)
**(**<15 days vs >30 days)	0.530	0.94 (0.79-1.13)
**(**15—30 days vs >30 days)	0.628	1.11 (0.75-1.63)
**Transfusion dependency at diagnosis**		
(yes vs no)	0.139	1.26 (0.93-1.71)
**Complex Karyotype**		
(yes vs no)	*<0.001*	2.30 (1.61-3.33)
**ELN risk category**		
Adverse risk vs low-intermediate risk	*<0.001*	1.78 (1.32-2.42)
**Type of HMA**		
(AZA vs DEC)	0.810	1.04 (0.74-1.15)
**Starting HMA dose**		
(reduced vs standard)	0.437	1.38 (0.61-3.12)
**BM blast percentage after 4th cycle**		
(≥30% vs <30%)	*0.036*	1.61 (1.03-2.51)
**Type of response after 4th cycle**		
(≥PR vs less than PR)	*<0.001*	0.50 (0.31-0.70)
**Type of best response**		
(≥PR vs less than PR)	*<0.001*	0.18 (0.08-0.37)
**Transfusion Independence**		
(yes vs no)	*<0.001*	0.39 (0.28-0.59)
**Treatment-related complication**		
(yes vs no)	0.700	1.15 (0.56-2.36)

Abbreviation: AML, acute myeloid leukemia; AZA, azacitidine; BM, bone marrow; BMI, body mass index; CCI, Charlson comorbidity index; CI, confidence interval; DEC, decitabine; ECOG PS, Eastern Cooperative Oncology Group Performance Status; eGFR, estimated glomerular filtration rate; ELN, European Leukemia Net; HMA, hypomethylating agents**;** HR= hazard ratio; OS, Overall Survival; PR, partial remission; s-AML, secondary AML

Supplemental Table S3. Univariate Cox Proportional Hazards Models for Event-free Survival

Variable	Predictive Factor	
	**p value**	**HR (95% CI)**
**Age at diagnosis**		
(≥80 vs 75-80 years)	*0.003*	1.61 (1.18-2.21)
**Sex**		
(female vs male)	0.553	0.92 (0.68-1.22)
**ECOG PS**		
(≥2 vs <2)	*0.053*	1.42 (0.99-2.03)
**Type of AML**		
(s-AML vs de novo AML)	0.005	1.53 (1.13-2.05)
**eGFR**		
(<30 vs ≥30 ml/min/1.73m^2)	0.198	1.30 (0.87-1.93)
**CCI**		
(≥3 vs <3)	*0.007*	1.67 (1.17-2.45)
**BMI at diagnosis**		
(<25 vs ≥25)	0.631	0.93 (0.70-1.25)
**BM blast percentage**		
(≥50% vs <50%)	*<0.001*	2.18 (1.59-2.98)
**Infectious at AML diagnosis**		
(no vs yes)	0.128	0.76 (0.54-1.08)
**Time from diagnosis to the start of therapy**		
(<15 days vs 15—30 days)	0.102	1.35 (0.94-1.94)
(<15 days vs >30 days)	0.759	0.97 (0.82-1.15)
(15—30 days vs >30 days)	0.251	0.80 (0.55-1.17)
**Transfusion dependency at diagnosis**		
(yes vs no)	0.393	1.14 (0.85-1.54)
**Complex Karyotype**		
(yes vs no)	*<0.001*	2.39 (1.66-3.42)
**ELN risk category**		
Adverse risk vs low-intermediate risk	*<0.001*	1.93 (1.44-2.59)
**Type of HMA**		
(AZA vs DEC)	0.947	0.98 (0.71-1.37)

**Starting HMA dose**		
(reduced vs standard)	0.593	1.23 (0.57-2.63)
**BM blast percentage after 4th cycle**		
(≥30% vs <30%)	0.071	1.50 (0.97-2.32)
**Type of response after 4th cycle**		
(≥PR vs less than PR)	*0.003*	0.55 (0.36-0.82)
**Type of best response**		
CR vs PR	*0.027*	0.51 (0.28-0.93)
CR vs SD	*<0.001*	0.37 (0.23-0.59)
PR vs SD	0.141	0.65 (0.37-1.15)
**Transfusion Independence**		
(yes vs no)	*<0.001*	0.16 (0.10-0.33)
**Treatment-related complication**		
(yes vs no)	0.870	1.06 (0.52-2.18)

Abbreviation: AML, acute myeloid leukemia; AZA, azacitidine; BM, bone marrow; BMI, body mass index; CCI, Charlson comorbidity index; CI, confidence interval; CR, complete remisison; DEC, decitabine; ECOG PS, Eastern Cooperative Oncology Group Performance Status; eGFR, estimated glomerular filtration rate; ELN, European Leukemia Net; HMA, hypomethylating agents**;** HR= hazard ratio; OS, Overall Survival; PR, partial remission; s-AML, secondary AML
